# Modifiable traits, healthy behaviours, and leukocyte telomere length: a population-based study in UK Biobank

**DOI:** 10.1016/S2666-7568(22)00072-1

**Published:** 2022-05

**Authors:** Vasiliki Bountziouka, Crispin Musicha, Elias Allara, Stephen Kaptoge, Qingning Wang, Emanuele Di Angelantonio, Adam S Butterworth, John R Thompson, John N Danesh, Angela M Wood, Christopher P Nelson, Veryan Codd, Nilesh J Samani

**Affiliations:** a Department of Cardiovascular Sciences, University of Leicester, Leicester, UK; b Department of Health Sciences, University of Leicester, Leicester, UK; c NIHR Leicester Biomedical Research Centre, Glenfield Hospital, Leicester, UK; d British Heart Foundation Cardiovascular Epidemiology Unit, Department of Public Health and Primary Care, National Institute for Health Research Blood Transplant Research Unit in Donor Health and Genomics, and British Heart Foundation Centre of Research Excellence, University of Cambridge, Cambridge, UK; e Health Data Research UK Cambridge, University of Cambridge, Cambridge, UK; f Health Data Science Centre, Human Technopole, Milan, Italy; g Wellcome Sanger Institute, Wellcome Genome Campus, Hinxton, UK

## Abstract

**Background:**

Telomere length is associated with risk of several age-related diseases and cancers. We aimed to investigate the extent to which telomere length might be modifiable through lifestyle and behaviour, and whether such modification has any clinical consequences.

**Methods:**

In this population-based study, we included participants from UK Biobank who had leukocyte telomere length (LTL) measurement, ethnicity, and white blood cell count data. We investigated associations of LTL with 117 potentially modifiable traits, as well as two indices of healthy behaviours incorporating between them smoking, physical activity, diet, maintenance of a healthy bodyweight, and alcohol intake, using both available and imputed data. To help interpretation, associations were summarised as the number of equivalent years of age-related change in LTL by dividing the trait β coefficients with the age β coefficient. We used mendelian randomisation to test causality of selected associations. We investigated whether the associations of LTL with 22 diseases were modified by the number of healthy behaviours and the extent to which the associations of more healthy behaviours with greater life expectancy and lower risk of coronary artery disease might be mediated through LTL.

**Findings:**

422 797 participants were available for the analysis (227 620 [53·8%] were women and 400 036 [94·6%] were White). 71 traits showed significant (p<4·27 × 10^–4^) associations with LTL but most were modest, equivalent to less than 1 year of age-related change in LTL. In multivariable analyses of 17 traits with stronger associations (equivalent to ≥2 years of age-related change in LTL), oily fish intake, educational attainment, and general health status retained a significant association of this magnitude, with walking pace and current smoking being additionally significant at this level of association in the imputed models. Mendelian randomisation analysis suggested that educational attainment and smoking behaviour causally affect LTL. Both indices of healthy behaviour were positively and linearly associated with LTL, with those with the most healthy behaviours having longer LTL equivalent to about 3·5 years of age-related change in LTL than those with the least heathy behaviours (p<0·001). However, healthy behaviours explained less than 0·2% of the total variation in LTL and did not significantly modify the association of LTL with risk of any of the diseases studied. Neither the association of more healthy behaviours on greater life expectancy or lower risk of coronary artery disease were substantially mediated through LTL.

**Interpretation:**

Although several potentially modifiable traits and healthy behaviours have a quantifiable association with LTL, at least some of which are likely to be causal, these effects are not of a sufficient magnitude to substantially alter the association between LTL and various diseases or life expectancy. Attempts to change telomere length through lifestyle or behavioural changes might not confer substantial clinical benefit.

**Funding:**

UK Medical Research Council, UK Biotechnology and Biological Sciences Research Council, and British Heart Foundation.

## Introduction

Telomeres, the cap structures at the ends of chromosomes, vary in their average length between individuals, with telomere length being an important determinant of cellular replicative capacity.^1^ Inter-individual variation in telomere length, most commonly measured in blood (leukocyte telomere length [LTL]) is a highly heritable trait, with estimates of heritability of about 0·70, and is present both at birth and throughout the life course.^2^, ^3^, ^4^ Other major correlates of LTL are age, sex, and ethnicity, with older age, male sex, and White ethnicity associated with shorter LTL.^5^ LTL is associated with a range of human biological traits, age-related diseases, and lifespan, with growing evidence that some of these associations are causal.^6^, ^7^, ^8^ However, its relationship with diseases is complex: shorter LTL is associated with increased risk of degenerative diseases, such as coronary artery disease, but decreased risk of several cancers.^6^, ^7^, ^8^

The relationship between LTL and disease risk has led to much interest in understanding whether an individual can potentially modify their LTL and, consequently, the associated risk of disease. At the cellular level, telomere length shortens in response to oxidative and inflammatory stress.^9^, ^10^ Many studies have therefore sought to explore the relationship between LTL and a range of potentially modifiable environmental and lifestyle factors associated with oxidative stress and inflammation, including smoking,^11^, ^12^ obesity,^11^, ^13^ diet,^14^, ^15^ and physical activity.^16^, ^17^ However, many of these studies have shown conflicting results, perhaps in part due to moderate sample sizes. Meta-analyses have strengthened the evidence of associations between smoking, and between higher body-mass index (BMI), and shorter LTL.^12^, ^13^ Lower physical activity is generally associated with shorter LTL, although studies remain inconsistent, potentially due to diversity of methods used in the assessment of physical activity.^16^ The relationship between LTL and dietary factors remains to be fully established, although evidence suggests that a Mediterranean diet is associated with longer LTL.^15^ Overall, the available studies suggest that healthy behaviours are potentially associated with longer LTL, whereas unhealthy behaviours are associated with shorter LTL. In addition, other traits and behaviours that affect LTL await discovery.

**Research in context**

**Evidence before this study**

Growing evidence that variation in leukocyte telomere length (LTL) is associated with risk of several age-related diseases (including coronary artery disease and several cancers) and overall life expectancy has sparked interest in whether LTL can be affected by lifestyle and behaviours. Previous studies have shown that LTL is associated with certain potentially modifiable factors such as smoking, body-mass index, diet, and physical activity. Overall, the available studies suggest that healthy behaviours are potentially associated with longer LTL, whereas unhealthy behaviours are associated with shorter LTL. However, a systematic analysis of such associations has not been undertaken, and whether they are causal is unknown. Furthermore, no studies have addressed the composite question of whether a healthy lifestyle overall is associated with longer LTL, and whether such an association has any clinical consequences.

**Added value of this study**

In this study, using LTL measurements that we had generated in UK Biobank, we investigated associations of LTL with 117 potentially modifiable traits, as well as two indices of healthy behaviours (incorporating smoking, physical activity, diet, maintenance of a healthy bodyweight, and alcohol intake) in up to 422 797 participants. 71 traits showed significant associations with LTL, but most were modest, equivalent to less than 1 year of age-related change in LTL. In multivariable analyses, five traits—oily fish intake, educational attainment, general health status, walking pace, and current smoking—retained a significant association equivalent to 2 years or longer of age-related change in LTL. Mendelian randomisation analysis suggested that educational attainment and smoking behaviour causally affect LTL. Both indices of healthy behaviour were positively and linearly associated with LTL. However, healthy behaviours only explained less than 0·2% of the total variation in LTL and did not significantly modify the association of LTL with risk of any of 22 diseases that are causally associated with LTL. Neither the association of more healthy behaviours on greater life expectancy or lower risk of coronary artery disease were substantially mediated through LTL.

**Implications of all the available evidence**

Although several potentially modifiable traits and healthy behaviours have a quantifiable association with LTL, at least some of which are likely to be causal, these effects are not of a sufficient magnitude to substantially alter the association between LTL and various diseases or life expectancy. Attempts to change telomere length through lifestyle or behavioural changes might not confer substantial clinical benefit. Other approaches are required to modify the association of telomere length with diseases.

UK Biobank, is a large, prospective study that recruited participants between 2006 and 2010, and has collected detailed and extensive information on lifestyle and behaviour.^18^ We have recently completed measurement of LTL in 474 074 participants in the UK Biobank.^5^ We therefore sought to use this new resource to undertake a comprehensive cross-sectional analysis to identify potentially modifiable factors that might influence LTL, and also to address the composite question of whether, and to what extent, healthy behaviour, defined using previously described indices,^19^, ^20^ is associated with longer LTL. In addition, we aimed to investigate whether healthy behaviour influences the association of LTL with diseases and to what extent the associations of more healthy behaviours with greater life expectancy and lower risk of coronary artery disease might be mediated through LTL.

## Methods

### Study design and participants

In this population-based analysis, we included participants with LTL measurements in UK Biobank, which recruited participants aged 40–69 years. We excluded genetically related samples (randomly excluding one from each pair based on a kinship coefficient of more than 0·088), samples with no genetic data, and samples that failed quality control. We also excluded participants who had no information on ethnicity or white blood cell count, which are both associated with LTL^5^ and were used together with age and sex to adjust the trait associations.

LTL was measured on DNA extracted from blood samples collected at baseline using an established quantitative PCR method and reported as a ratio of the telomere repeat number to a single-copy gene. The measurements were log_e_ transformed to approximate the normal distribution and were then transformed to Z-standardised values (UK Biobank field code 22192) to facilitate comparison with other datasets. Further details of the LTL measurements and their quality control have been reported elsewhere.^5^

UK Biobank received approval from the North West Centre for Research Ethics Committee (11/NW/0382). The use of data presented in this paper was approved by the Access Committee of UK Biobank under application number 6077.

### Modifiable traits selection

We considered characteristics recorded at baseline in at least 50 000 UK Biobank participants and identified 117 potentially modifiable traits, including three derived traits: waist-to-hip ratio, pulse pressure, and alcohol intake (appendix pp 14–20). These traits were grouped under 12 major categories, representing (alphabetically): alcohol intake (four traits), anthropometry (12), blood biochemistry (13), cardiovascular (six), chronobiology (five), dietary intake (34), early life and sexual health (five), general health (11), physical activity (five), psychosocial (13), smoking (two), and socioeconomic status (seven; appendix pp 14–20). Although the extent to which some of the traits are modifiable is debatable, we chose to be inclusive in our selection to evaluate as comprehensively as possible traits that could potentially influence LTL.

### Healthy behaviour indices

We generated our primary healthy behaviour index according to a previously reported method.^19^ Healthy behaviour was considered as the absence of current or previous smoking; engagement with physical activities that require the expenditure of more than 735 metabolic equivalents (MET) per h per week; maintenance of a healthy bodyweight assessed with a BMI in the range of 18·5–24·9 kg/m^2^; adherence to a healthy diet; and moderate alcohol consumption (appendix pp 3, 21). Adherence to a healthy diet is characterised by a higher consumption of fruits, nuts, vegetables, whole grains, fish, and dairy products, and a lower consumption of refined grains, processed meats, unprocessed red meats, and sugar-sweetened beverages.^21^ A dietary index was created from the cumulative sum of the level of consumption of these components (theoretical range of 0–7). Further details are provided in the appendix (p 3). Participants with scores greater than or equal to 4 in the diet index were considered to follow a healthy diet. For the assessment of alcohol consumption, we considered participants’ self-reported weekly and monthly intake of different types of alcoholic beverages and converted this into grams of alcohol per day (appendix p 3). Moderate alcohol intake was then considered as 5–15 g per day for women and 5–30 g per day for men, respectively equivalent to one or fewer and two or fewer small wine glasses of 12% alcohol by volume.^19^ Alcohol intake for participants who reported current non-drinking (including formal drinkers) was assumed to be 0 g per day of alcohol. For each behavioural component, a score of 1 was given if the participant met the criterion for healthy behaviour, or 0 otherwise. The scores were then summed to create our primary healthy behaviour index that ranged from 0 (no healthy behaviours) to 5 (adherence to all five healthy behaviours).

To test the robustness of any association of LTL with healthy behaviour, we also created a second healthy behaviour index based on another index that has been used previously.^20^ In this index, healthy behaviours were considered to be an absence of current smoking; moderate or vigorous levels of physical activity; maintenance of a healthy bodyweight assessed as a BMI of less than 30 kg/m^2^; and adherence to a healthy diet (appendix p 21). Alcohol intake was not included (appendix p 21). Participants were assigned a score of 1 if they met the criterion of a healthy behaviour component. The sum of these four scores provided a range from 0 (no healthy behaviours) to 4 (adhering to all four healthy behaviour components). A comparison of the two healthy behaviour indices and detailed scoring for each are shown in the appendix (p 21).

### Outcomes

To evaluate the potential clinical relevance of our findings, we did two types of analyses. First, we examined whether the association of LTL with 22 diseases, for which we have previously found evidence of a potential causal association in UK Biobank through mendelian randomisation analysis,^8^ differs according to the number of healthy behaviours, using the primary healthy behaviour index. Second, as we have previously shown that longer LTL is associated with greater life expectancy and lower risk of coronary artery disease,^8^ and healthy behaviours also affect these phenotypes, we calculated the proportion of any association of primary healthy behaviour index on life expectancy and risk of coronary artery disease that might be mediated through LTL. Further details of these analyses are provided in the appendix (pp 5–6, 11–13, 31–33).

### Statistical analysis

We based our main analysis on available data for each individual trait and each healthy behaviour index. In addition, to assess the effect of missing trait values, particularly for the multivariable analysis, we repeated our analyses using multiple imputation by chained equations^22^ with ten imputed datasets. The imputation models included all modifiable traits, medical conditions, age, sex, ethnicity, and white blood cell count, and we specified linear, logistic, and multinomial regression imputation models for continuous, binary, and categorical traits, respectively. To ensure convergence, we performed ten iterations for each imputed dataset.^22^ We compared the Monte Carlo error against the SEs of the estimated β coefficients (parameters) of the various traits to evaluate the performance of the imputation.

To identify single traits significantly associated with LTL, we fitted a multivariable linear regression model for each trait with Z-standardised LTL as the response variable, adjusting for age, sex, ethnicity, and white blood cell count (because these variables have been shown to be associated with LTL^5^). In these analyses, continuous traits were first winsorised at the 0·5% and 99·5% percentile values to exclude extreme outliers. Traits were log_e_ transformed where appropriate after graphically checking their distributions, before being scaled to the standardised normal distribution to aid interpretation. For continuous traits, results are shown as β coefficients per 1 SD increase in the trait, whereas for binary traits results are presented as β coefficients from a comparison of yes versus no status. Significance for the trait analyses was set at a Bonferroni-corrected threshold of p values of less than 4·27 × 10^–4^ to account for the number of traits analysed. To aid interpretation, we calculated the association with each trait in terms of number of equivalent years of age-related change in LTL, by dividing the β coefficient for the trait by the absolute value of the β coefficient for the age-related change in LTL (–0·023 per year).^5^ Because of potential correlation between traits associated with LTL, to identify those that had the strongest independent associations with LTL, we also fitted a multivariable linear regression model for multiple traits that were Bonferroni significant and had an association with LTL equivalent to an age effect on LTL of 2 years or longer in the analysis on the available data.

To examine the association between the two healthy behaviour indices and LTL, we also used linear regression models. In the base model, we analysed the association between the healthy behaviour index and LTL, adjusting for age, sex, ethnicity, and white blood cell count. Because behaviours might be affected by the presence of disease, we next adjusted for the presence of several self-reported chronic medical conditions that were diagnosed by a doctor, including diabetes (UK Biobank field code 2443), cancer (2453), and vascular disease and hypertension (both derived from field 6150) at the time of enrolment into UK Biobank (adjusted model). Finally, to account for potential confounding by other modifiable traits that were not directly considered as part of the healthy behaviour indices, we additionally adjusted for the most significant traits as proxies for each category (final model; appendix pp 14–20). Specifically, we adjusted for educational attainment as a proxy for socioeconomic status, insomnia as a proxy for chronobiology, fed-up feelings as proxy for psychosocial wellbeing, and LDL cholesterol, C-reactive protein, and estimated glomerular filtration rate (eGFR; calculated with the CKD-EPI equation) as representatives of biochemical traits. p values were estimated with the Jonckheere-Terpstra test for trend for both continuous and categorical traits.

To investigate the causality and directionality of traits that showed the strongest association with LTL in both the univariable and multivariable analyses, we did a bidirectional mendelian randomisation analysis that under certain assumptions intends to estimate causal effects, using large-scale genome-wide association study (GWAS) datasets, for LTL and the traits with the strongest association (educational attainment and smoking).^8^, ^23^, ^24^ For educational attainment, to assess whether the direction of effect was from education to LTL, we used genetic variants that were associated with number of years of schooling in a GWAS of 1·1 million individuals^23^ as mendelian randomisation instruments and examined their association with LTL in the LTL GWAS dataset.^8^ For smoking, we used genetic variants associated with two smoking phenotypes: initiation of regular smoking (a binary phenotype indicating whether an individual had ever smoked regularly [current or former] or not), and smoking intensity (cigarettes smoked per day for both current and former smokers).^24^ In each case, to examine the converse possibility—ie, that the direction of association is from LTL to educational attainment and smoking—we used genetic variants that we have recently reported to be associated with LTL in UK Biobank,^8^ and studied their associations with educational attainment and smoking behaviour in the respective GWASs. Further details of the mendelian randomisation analysis, its assumptions, the number of genetic variants used in each analysis, and the proportion of trait variances explained by these are provided in the appendix (p 4). Data analyses for observational data were done with Stata (version 17). All mendelian randomisation analyses and imputation models were performed in R (version 3.1.6).

### Role of the funding source

The funders of the study had no role in study design, data collection, data analysis, data interpretation, writing of the report, or the decision to submit for publication.

## Results

422 797 participants were available for the analysis (table 1; appendix p 8). The participants had a mean age of 56·6 years (SD 8·0), with more women (227 620 [53·8%]) than men, and were predominantly of White ethnicity (400 036 [94·6%]).

**Table 1:** Selected characteristics of the UK Biobank participants analysed

		**UK Biobank field**	**Participants (n=422 797)**
Z-standardised telomere length		22 192	0·0 (1·0)
Sex		31	..
	Male	..	195 177 (46·2%)
	Female	..	227 620 (53·8%)
Age at recruitment, years		54	56·6 (8·0)
Ethnicity		21 000	..
	White	..	400 036 (94·6%)
	Mixed	..	2518 (0·6%)
	Asian	..	8355 (2·0%)
	Black	..	6587 (1·6%)
	Chinese	..	1373 (0·3%)
	Other	..	3928 (0·9%)
White blood cell count, 10^9^ cells per L		30 000	6·9 (1·74)
Townsend deprivation index		189	−1·32 (3·08; n=422 260)
Body-mass index, kg/m^2^		23 104	27·40 (4·69; n=421 165)
Diabetes diagnosed by doctor		2443	22 117/421 520 (5·3%)
Cancer diagnosed by doctor		2443	32 068/421 393 (7·6%)
Vascular disease diagnosed by doctor		6150	24 428/421 828 (5·8%)
Hypertension diagnosed by doctor		6150	114 549/421 828 (27·2%)
Diastolic blood pressure, mm Hg		4079	82·2 (10·0; n=399 424)
Systolic blood pressure, mm Hg		4080	137·8 (18·4; n=399 421)
LDL cholesterol, mmol/L		30 780	3·6 (0·9; n=404 553)
C-reactive protein, mg/L		30 710	2·5 (3·7; n=404 439)
Estimated glomerular filtration rate, mg/dL (from creatinine)		30 700	77·4 (75·1; n=405 094)

Data are mean (SD), n (%), or n/N (%). UK Biobank field=UK Biobank code from which the trait data are derived.

**Figure 1:**
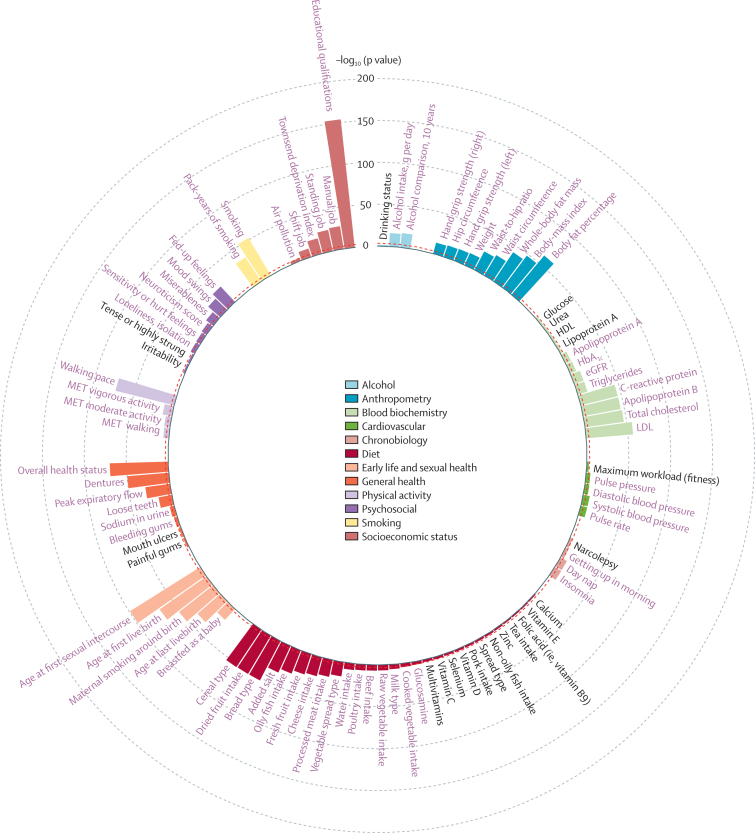
A circular plot showing traits nominally associated with leukocyte telomere length For each trait, the p value shown is from a multivariable linear regression model adjusted for age, sex, ethnicity, and white blood cell count. For categorical traits, the global p value from a likelihood ratio test is shown. Bonferroni significant traits (p<4·27 × 10^–4^) are in purple text. Nominally significant traits (p<0·05) are in black text. Non-significant traits (p>0·05) are shown in the appendix (pp 14–20). eGFR=estimated glomerular filtration rate. HbA_1c_=glycated haemoglobin. MET=metabolic equivalents.

**Figure 2:**
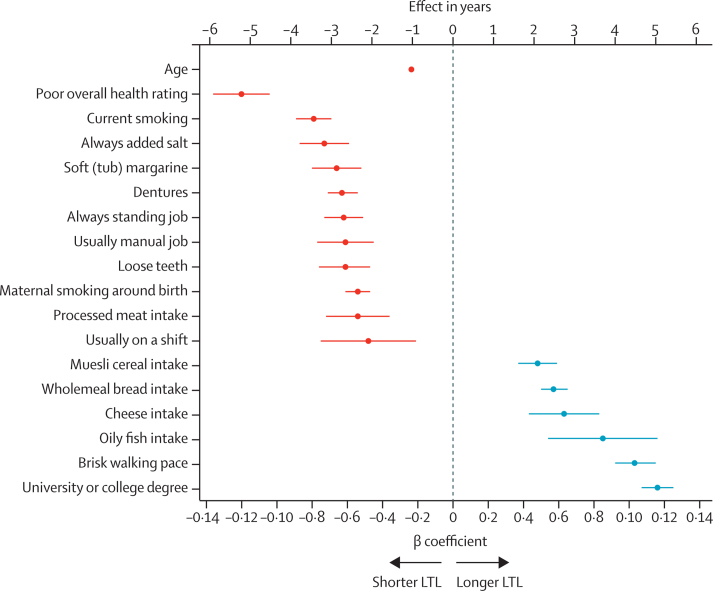
Individual traits most strongly associated with LTL The traits are shown on the y-axis and the β coefficients for the associations LTL on the x-axis. Error bars indicate 95% CIs. Effect in years is the ratio of the trait β coefficient and the absolute value of the age β coefficient (–0·023). Continuous traits are estimated for a 1 SD increase in the trait. Binary traits compare yes with no. Poor overall health rating is compared with excellent. Current smoking is compared with never. Always added salt is compared with never or rarely. Soft (tub) margarine is compared with olive-oil spread. Always standing job or usually manual job is compared with never or rarely. Processed meat, cheese, or oily fish intake (five or more times per week) is compared with never. Usually on a shift is compared with never. Muesli cereal intake is compared with other types. Wholemeal bread intake is compared with white bread. Brisk walking pace is compared with slow. University or college degree is compared with no qualifications. LTL=leukocyte telomere length.

Of the 117 potentially modifiable individual traits analysed, 71 (60·7%) showed a significant association with LTL after adjusting for multiple testing (p<4·27 × 10^–4^; figure 1; appendix pp 9, 14–20). The findings from the multiple imputation analysis were highly concordant (appendix pp 14–20). For many of the traits, although the associations were significant, the β coefficients were small and equivalent to less than 1 year of age-related change in LTL (appendix pp 14–20). However, for 17 traits, the associations were more substantial (≥2 years of age-related change in LTL) per 1 SD change in the trait or group contrast (figure 2). As some of these traits might be correlated with each other, a multivariable regression analysis was done to identify those with the strongest independent associations. From the available data, this analysis was only possible in 84 462 participants with data on all 17 traits. The univariate associations for these traits in this subset of participants were similar to those seen in the fully available data for each trait (appendix pp 22–23). In the multivariable analysis of the available data, three traits retained a significant association equivalent to 2 years or longer age-related change in LTL (appendix pp 22–23). Compared with participants who had no intake of oily fish, those who had an intake of oily fish of two to four times a week had longer LTL equivalent to 2·4 years of age-related change in LTL (p=5·5 × 10^–5^); compared with participants who reported excellent overall health status, those with poor overall health status on average had shorter LTL equivalent to 4·0 years of age-related change in LTL (p=4·1 × 10^–4^); and compared with participants whose education was at the level of below end of secondary school, those who attained degree-level education had a longer LTL equivalent to 2·8 years of age-related change in LTL years (p=1·6 × 10^–6^). For all three traits, we saw a gradient of difference in LTL across more granular levels of the trait, adding confidence to the findings (appendix pp 22–23). Furthermore, in the analysis of the multivariable multiple imputation analysis for these 17 traits, both intake of oily fish and educational attainment showed very similar associations to those seen with the available data (appendix pp 22–23). The magnitude of the association with overall health status became less strong (equivalent to 2·0 years of age-related change in LTL) but remained significant (p=4·2 × 10^–7^), whereas the associations with brisk walking pace with longer LTL (equivalent to 2·2 years of age-related change in LTL, p=3·3 × 10^–14^) and current smoking with shorter LTL (equivalent to 2·0 years of age-related change in LTL, p=3·5 × 10^–17^) became stronger (appendix pp 22–24). Educational attainment is associated with a wide range of other characteristics, including social deprivation (which is linked to socioeconomic status), dietary and drinking habits, physical activity, and BMI. Adjusting for these potential confounders marginally attenuated the association of educational attainment with LTL, which remained highly significant (appendix p 24).

329 907 participants had complete information to calculate the primary healthy behaviour index (appendix p 8). For the primary healthy behaviour index, 14 576 (4·4%) participants had a score of 0 for healthy behaviour, 69 082 (20·9%) had a score of 1, 114 086 (34·6%) had a score of 2, 90 985 (27·6%) had a score of 3, 35 752 (10·8%) had a score of 4, and 5426 (1·6%) had a score of 5 (table 2). Although significant, there was no substantial difference in the mean age of participants across the scores and there was only minor variation in the proportion of women (table 2). However, several social and clinical characteristics showed a substantial gradient in prevalence from participants with no healthy behaviours to those with five healthy behaviours. This gradient was further reflected in the prevalence of several diseases. For example, the prevalence of diabetes was 11·1% among participants with a score of 0 compared with 1·5% in those with a score of 5. Likewise, the prevalence of hypertension and cardiovascular disease also showed around a three times difference between these groups (table 2).

**Table 2:** Participant demographics partitioned by number of healthy behaviours for the primary health behaviour index (n=329 907)

		**No healthy behaviours**	**One healthy behaviour**	**Two healthy behaviours**	**Three healthy behaviours**	**Four healthy behaviours**	**Five healthy behaviours**	**p value**
Participants		14 576 (4·4)	69 082 (20·9)	114 086 (34·6)	90 985 (27·6)	35 752 (10·8)	5426 (1·6)	..
Z-standardised leukocyte log_e_ telomere length		−0·07 (1·00)	−0·05 (1·00)	0·00 (1·00)	0·04 (1·00)	0·08 (0·98)	0·09 (1·01)	8·80 × 10^−135^
Age, years		56·9 (7·7)	56·7 (7·9)	56·4 (8·1)	56·0 (8·1)	55·8 (8·2)	56·2 (8·1)	9·77 × 10^−106^
Sex		..	..	..	..	..	..	7·06 × 10^−89^
	Female	7117 (48·8%)	33 944 (49·1%)	58 721 (51·5%)	48 278 (53·1%)	19 641 (54·9%)	2729 (50·3%)	..
	Male	7459 (51·2%)	35 138 (50·9%)	55 365 (48·5%)	42 707 (46·1%)	16 111 (45·1%)	2697 (49·7%)	..
Ethnicity		..	..	..	..	..	..	1·07 × 10^−34^
	White	13 985 (96·0%)	65 641 (95·0%)	107 867 (94·6%)	86 790 (95·4%)	34 641 (96·9%)	5308 (97·8%)	..
	Mixed	82 (0·6%)	488 (0·7%)	695 (0·6%)	490 (0·5%)	139 (0·4%)	24 (0·4%)	..
	Asian	194 (1·3%)	1206 (1·75%)	2210 (1·9%)	1555 (1·7%)	455 (1·3%)	47 (0·9%)	..
	Black	156 (1·1%)	1015 (1·5%)	1910 (1·7%)	1092 (1·2%)	231 (0·65%)	20 (0·4%)	..
	Chinese	10 (0·1%)	128 (0·2%)	361 (0·3%)	384 (0·4%)	100 (0·28%)	5 (0·1%)	..
	Other	149 (1·0%)	604 (0·9%)	1043 (0·9%)	674 (0·7%)	186 (0·5%)	22 (0·4%)	..
White blood cell count, 10^9^ cells per L		7·52 (1·91)	7·18 (1·80)	6·90 (1·71)	6·61 (1·61)	6·37 (1·53)	6·20 (1·50)	<1·00 × 10^−300^
Highest education		..	..	..	..	..	..	<1·00 × 10^−300^
	None	3010 (20·8%)	12 632 (18·4%)	17 247 (15·2%)	10 463 (11·6%)	2992 (8·4%)	339 (6·28%)	..
	O levels or CSE	2681 (18·5%)	12 301 (17·9%)	19 178 (16·9%)	14 199 (15·7%)	4883 (13·7%)	628 (11·6%)	..
	A levels, NVQ, other	4921 (34·0%)	23 561 (34·3%)	39 050 (34·5%)	30 031 (33·2%)	10 856 (30·5%)	1530 (28·3%)	..
	Degree	3866 (26·7%)	20 122 (29·3%)	37 803 (33·4%)	35 757 (39·5%)	16 868 (47·4%)	2904 (53·8%)	..
Insomnia		..	..	..	..	..	..	<1·00 × 10^−300^
	Never or rarely	2944 (20·2%)	15 830 (22·9%)	28 646 (25·1%)	24 383 (26·8%)	10 233 (28·6%)	1614 (29·8%)	..
	Sometimes	6383 (43·8%)	32 117 (46·5%)	54 068 (47·4%)	44 052 (48·4%)	17 319 (48·5%)	2620 (48·3%)	..
	Usually	5242 (36·0%)	21 099 (30·6%)	31 330 (27·5%)	22 519 (24·8%)	8186 (22·9%)	1189 (21·9%)	..
Fed-up feelings		7370 (51·4%)	30 178 (44·4%)	44 511 (39·6%)	31 560 (35·3%)	11 058 (31·4%)	1480 (27·6%)	<1·00 × 10^−300^
LDL cholesterol, mmol/L		3·6 (0·9)	3·59 (0·9)	3·6 (0·9)	3·6 (0·8)	3·5 (0·8)	3·4 (0·8)	3·68 × 10^−69^
C-reactive protein, mg/L		3·9 (4·7)	3·1 (4·0)	2·6 (3·6)	2·0 (3·1)	1·5 (2·9)	1·3 (2·6)	<1·00 × 10^−300^
Estimated glomerular filtration rate, mg/dL		71·2 (76·2)	71·2 (75·8)	74·0 (75·3)	76·2 (74·5)	78·6 (73·6)	71·9 (73·3)	8·11 × 10^−62^
Diabetes		1618 (11·1%)	4907 (7·1%)	6051 (5·3%)	3057 (3·4%)	697 (1·95%)	80 (1·5%)	<1·00 × 10^−300^
Cancer		1257 (8·7%)	5339 (7·8%)	8602 (7·7 %)	6491 (7·2 %)	2566 (7·2 %)	383 (7·1%)	1·99 × 10^−11^
Hypertension		5563 (38·2%)	22 847 (33·1%)	32 234 (28·3%)	19 927 (21·9%)	5728 (16·0%)	780 (14·4%)	<1·00 × 10^−300^
Vascular disease		1541 (10·6%)	5225 (7·6%)	6532 (5·7%)	3590 (4·0%)	1081 (3·0%)	161 (3·0%)	<1·00 × 10^−300^

Data are mean (SD) or n (%). Diseases are self-reported as diagnosed by doctor. p values were estimated with the Jonckheere-Terpstra test for trend for both continuous and categorical traits.

**Figure 3:**
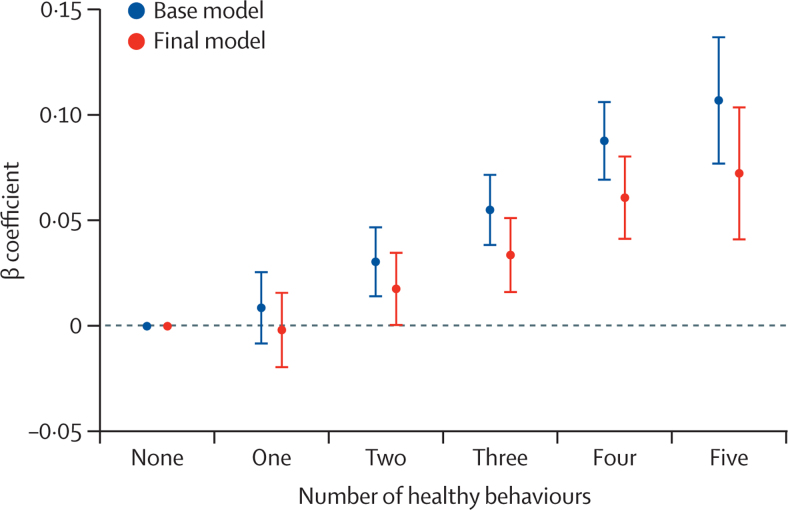
Association between the number of components of the primary healthy behaviour index and leukocyte telomere length In the base model adjustments were made for age, sex, ethnicity, and white blood cell count, and in the final model additional adjustments were made for self-reported diagnosed by doctor of chronic medical conditions (diabetes, cancer, hypertension, and vascular disease), insomnia, fed-up feelings, LDL cholesterol, C-reactive protein, estimated glomerular filtration rate, and educational attainment. Error bars represent the 95% CIs.

For the primary healthy behaviour index, in the base model (adjusted for age, sex, ethnicity, and white blood cell count), participants with adherence to all healthy behaviours (score 5) had significantly longer LTL on average (β coefficient=0·107, 95% CI 0·077–0·137, p=5·1 × 10^–12^) than those with no healthy behaviours (score 0; appendix p 25), equivalent to 4·6 years (95% CI 3·3–5·9) in age-related change in LTL. Adjusting the model for the presence of chronic health conditions (diabetes, cancer, hypertension, and vascular diseases) did not alter the association (β coefficient=0·103, 95% CI 0·072–0·133, p=3·9 × 10^–11^ appendix p 25). The association was modestly attenuated after further adjustments for higher educational level, insomnia, fed-up feelings, LDL cholesterol, C-reactive protein, and eGFR, but remained significant (β coefficient=0·072; 95% CI 0·041–0·104, p=7·7 × 10^–6^), equivalent to 3·1 years (95% CI 1·8–4·5) of age-related change in LTL (final model; appendix p 25). Across the primary healthy behaviour index score, there was a consistently additive dose–response association of greater healthy behaviours with longer LTL (figure 3). The findings were very similar from the multiple imputation analysis across all models (appendix p 25). For example, in the final model, for the comparison of five healthy behaviours versus none, the β coefficient was 0·074 (95% CI 0·046–0·103, p=3·39 × 10^–7^) for the imputed data (appendix p 25). Overall, the primary healthy behaviour index explained 0·180% (95% CI 0·179–0·181) of the variation in LTL.

331 658 participants had full information to calculate the second healthy behaviour index (appendix p 8). Despite the composition of the second healthy behaviour index being different (appendix p 21), and the proportion of participants across the scores being very different to that for the primary healthy behaviour index (appendix pp 26–27), we found the same pattern of positive linear association of greater adherence to healthy behaviours with longer LTL (appendix p 28). In the base model for the second healthy behaviour index including participants with available data, the β coefficient was 0·117 (95% CI 0·074–0·160, p=1·1 × 10^–7^) for participants with a score of 4 compared with a score of 0, equivalent to 5·1 years (95% CI 3·2–6·9) of age-related change in LTL, and in the final model it was 0·084 (0·039–0·129, p=2·9 × 10^–4^), equivalent to 3·7 years (1·7–5·6) of age-related change in LTL, again with highly concordant findings in the fully imputed data (appendix p 28).

To exclude the possibility that prevalent cancers or treatments for cancers might affect LTL, we repeated the analyses after excluding 40 165 individuals who, at recruitment, reported a history of one or more of 19 different cancer types (solid and blood; appendix p 29). Exclusion of these individuals did not alter the association of either the healthy behaviour indices or individual traits with LTL (appendix p 29). We also investigated whether the associations of the healthy behaviour indices, educational attainment, walking pace, fish intake, smoking status, and health differed by age or sex. In general, there were no major interactions (appendix p 10). However, the association of educational attainment and smoking status with LTL appeared to be less steep in participants aged 60–70 years than in those aged 40–49 years and 50–59 years, and that of oily fish intake with LTL was less steep in women than in men (appendix p 10).

**Figure 4:**
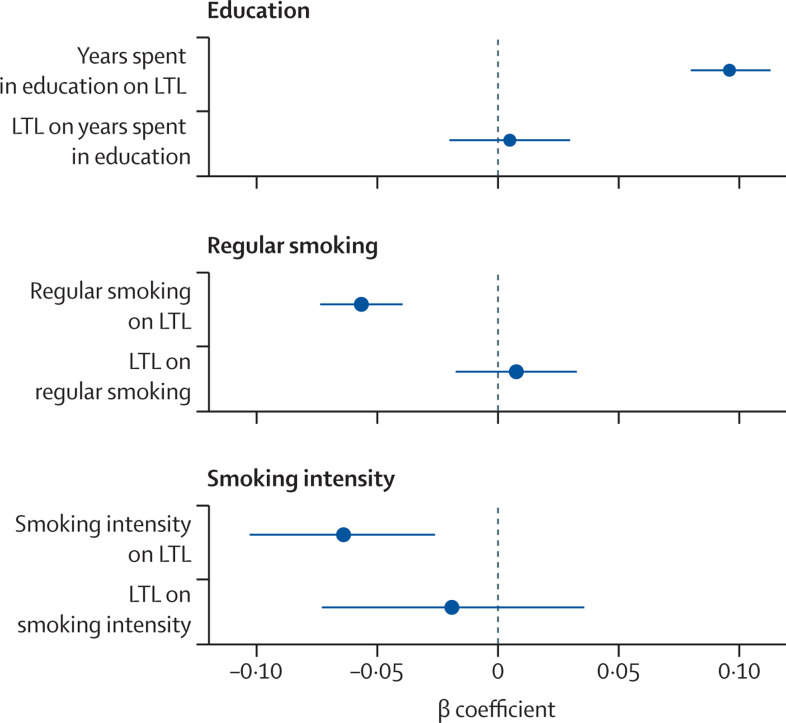
Mendelian randomisation analysis The plots show the results of the bidirectional mendelian randomisation analysis of the associations between LTL and years spent in education; initiation of regular smoking; and smoking intensity. Regression coefficients were derived from the inverse-weighted variance mendelian randomisation method. Error bars represent the 95% CIs. LTL=leukocyte telomere length.

In the mendelian randomisation analysis, there was a positive association between number of years spent in education and LTL. 1-year increase in the number of genetically determined years spent in education was associated with longer LTL, equivalent to 4·2 years (95% CI 3·5–4·9) of age-related change in LTL (p=3·19 × 10^–30^ figure 4; appendix p 30). There was no evidence of pleiotropy (mendelian randomisation Egger p=0·15) and other mendelian randomisation methods supported the findings (appendix p 30). The inverse mendelian randomisation analysis showed no evidence that LTL is causally associated with number of years spent in education (figure 4; appendix p 30). Similarly, the mendelian randomisation analysis supported a casual association of smoking behaviour with LTL with no evidence of reverse causation (figure 4; appendix p 30). A history of genetically determined smoking (current or former) was associated with shorter LTL equivalent to 2·5 years (95% CI 1·7–3·2) of age-related change in LTL (p=1·4 × 10^–10^; figure 4; appendix p 30), while 1 SD genetically determined increase in cigarettes smoked per day was associated with shorter LTL equivalent to 2·8 years (95% CI 1·1–4·5) of age-related change in LTL (p=0·0010; figure 4; appendix p 30).

Of 22 disorders for which we had previously seen concordant genetic and observational association with LTL, a possible interaction between healthy behaviour index and LTL was seen for one disease, sarcoma. Participants with four or five healthy behaviours had an inverse association between LTL and risk of sarcoma, whereas those with lower scores for healthy behaviours had a positive association (overall p_interaction_=0**·**0063); appendix pp 11, 31–33). However, when accounting for the number of tests performed, this was not significant (Bonferroni p=1·14 × 10^–3^), and there were 24 incident cases among participants with four or five healthy behaviours, necessitating caution in interpreting this finding.

A higher primary healthy behaviour index score was associated with longer life expectancy in both men and women from 40 years to older than 85 years (appendix p 12). At 40 years of age, men with a primary healthy behaviour index score of 5 had on average 10·4 years (95% CI 7·5–13·3) longer life expectancy than men with a score of 0. Corresponding estimates in women were 9·4 years (95% CI 6·2–12·6) longer life expectancy. Neither of these associations were significantly changed by adjustment for LTL (appendix p 12).

Each unit higher primary healthy behaviour index was associated with a 21% (95% CI 20–22) lower risk of coronary artery disease. The proportion of this risk that was mediated through LTL, although significant, was small (0·49%, 95% CI 0·33–0·65, p=2·8 × 10^–9^; appendix p 13). The findings were similar for the second healthy behaviour index (appendix p 13).

## Discussion

In the past two decades, compelling evidence has emerged that variation in telomere length affects multiple physiological traits as well as risk of several diseases. This finding has raised two important and hitherto unresolved questions: first, the extent to which telomere length might be modifiable through lifestyle factors and specific behaviours and, second, whether any such effect has important clinical consequences. In this study, by generating LTL measurements at a large scale in UK Biobank and using the extensive information on lifestyle, behaviour, and disease outcomes that have been collected on participants, we provide important new insights into these two questions.

The quantitative PCR method used to measure LTL in our study, although high throughput, provides a relative rather than absolute estimate of LTL, unlike other methods such as Southern blotting. Therefore, to help with interpretation of the biological and clinical significance of the observed associations, we converted these into number of equivalent years of age-related change in LTL calculated in the same cohort. In other studies using Southern blotting, change in LTL with age has been estimated at about 0·03 kb per year.^2^ Therefore, 1 year equivalent of age-related change in LTL is approximately equal to around 30 bp difference in LTL. To further contextualise this, in adults, average LTL has been estimated by Southern blotting to be between 7–11 kb.^1^, ^2^

In single-trait analysis of 117 potentially modifiable traits, we found demonstrable associations of LTL with many traits. Quantitatively, the associations were generally small and equivalent to less than 1 year of age-associated change in LTL. However, 17 traits showed stronger association equivalent to 2 years or longer of age-related change in LTL. In a multivariable analysis of these traits in the subset of participants in whom data were available on all of these traits, three remained significant and retained the larger effect sizes equivalent to 2 years or longer age-related change in LTL: oily fish intake, educational attainment, and general health status. Similar associations for these traits were seen in the multiple imputation analysis, which additionally showed significant associations with brisk walking pace and with current smoking (appendix pp 22–23).

The association of higher oily fish intake with longer LTL is notable because a previous longitudinal study showed that higher intake of marine omega-3 fatty acids was independently associated with a lower rate of telomere shortening over 5 years,^25^ suggesting a possible causal association. Using mendelian randomisation analysis, we have recently reported that the association of brisk walking pace with longer LTL is also likely to be causal.^26^ Additionally, in this study, our mendelian randomisation analysis of educational attainment and smoking behaviour suggests that the associations of these traits with LTL are also likely to be causal. Although the exact phenotypes used to identify the genetic variants in the GWAS analyses of education (number of years in schooling) and smoking behaviours (smoking initiation and smoking intensity) are not identical to the phenotypes available for these traits in UK Biobank and analysed here (educational attainment and current smoking), in both cases the different phenotypes are likely to be highly correlated. For smoking, the finding that both genetically determined smoking initiation and higher smoking intensity were associated with LTL provides further support for a causal association. Furthermore, we found no genetic evidence that LTL affects the duration of time in education or smoking behaviour to support the alternative explanation for the observed association between these traits and LTL. In aggregate, our findings indicate that at least some of the observed associations between the traits studied and LTL are likely to be causal. The precise mechanisms by which these traits affect LTL remain to be elucidated. For educational attainment, we found no evidence that the mechanism relates to other characteristics that are related to education, including socioeconomic status or lifestyle.

Although the associations of individual traits with LTL are interesting, perhaps the more important question from a clinical perspective is whether particular forms of healthy behaviour are associated with LTL, either individually or in aggregate. We calculated two previously reported indices of such behaviours, each comprising evaluation of smoking, physical activity, diet, and maintenance of a healthy bodyweight and, in one index, alcohol intake. Although the criteria for each behaviour in the two indices partitioned participants differently, we saw a similar pattern of association with LTL with both indices. In each case, the healthy behaviour score was positively and linearly associated with longer LTL. The finding was consistent between the analysis on participants with available data and the analysis on all participants after imputation of missing trait values. The association was not attenuated by the presence of several common chronic conditions (cancer, diabetes, hypertension, and vascular diseases), which might have altered behaviours and was only modestly attenuated by adjustment for other factors not included in the indices that differed between healthy behaviour groups. Notably, although C-reactive protein concentrations varied between the healthy behaviour index groups, being higher in participants with fewer healthy behaviours, adjustment for C-reactive protein concentration did not remove the association, suggesting that any effect on systemic inflammation might only explain part of the association between healthy behaviours and LTL.

Despite the reproducible association of healthy behaviours with LTL, the amount of inter-individual variation explained by such behaviours is very modest, accounting for less than 0·2% of such variation. To put this into context, age, sex, ethnicity, and white blood cell count together explain about 5·5% of variance in LTL.^5^ LTL is largely genetically determined, with heritability estimates of about 0·70.^2^, ^3^ The low variance explained by health behaviours probably explains why we did not see a significant influence of such behaviours on the association between LTL and various diseases. Similarly, although a higher healthy behaviour index score and longer LTL are both associated with greater life expectancy and lower risk of coronary artery disease,^8^ we found no evidence that a substantial proportion of the association of the healthy behaviour index with either of these is mediated through LTL.

Our findings have several individual-level and population-level implications. They demonstrate that telomere length might be modifiable by lifestyle and behaviour. However, the quantitative effects of these healthy behaviours are small compared with the much larger genetically driven variation in LTL between individuals. As such, attempts to modify the association between LTL and risk of various diseases through adoption of healthy behaviours or targeting a particular modifiable trait are likely to have modest effects at best. Furthermore, any ambition to target telomere length therapeutically needs to take into account that both shorter and longer telomeres are associated with risk of specific diseases. Therefore, developing a better understanding of the mechanisms by which variation in telomere length affects risk of individual diseases and then selectively targeting these mechanisms might be a better approach. For example, there is evidence that variation in telomere length affects the development of clonal haematopoiesis of indeterminate potential (CHIP), which in turn is associated with risk of coronary artery disease.^27^ Therefore, targeting the mechanisms through which CHIP affects coronary artery disease risk could, at least partly, attenuate the association of LTL with risk of coronary artery disease.

The strengths of our study include its large scale and the detailed phenotyping available in UK Biobank to enable a comprehensive and robust analysis of the association between modifiable traits and behaviours and LTL. However, as with any cross-sectional analysis, caution is necessary about making causal inferences, despite supportive genetic analysis. Furthermore, because longitudinal telomere measurements were not available, we are unable to assess whether within-individual changes in LTL with age are affected by modifiable traits and behaviours and whether these have clinical consequences. Additionally, our estimates of age-related changes in LTL are based on cross-sectional estimates and have been derived within the UK Biobank cohort, which might not be directly comparable to other studies. Finally, UK Biobank is under-represented in non-White ethnicities, and there is evidence that the cohort recruited participants that were healthier than the general population.^28^ It is therefore possible that the magnitude of the associations of modifiable traits and healthy behaviours with LTL might vary in other populations, and further studies are necessary.

In summary, we show that several potentially modifiable traits as well as healthy behaviours have a quantifiable association with LTL. At least in some cases, this association is likely to be causal. However, the effect on LTL is modest, and although modifying these traits and behaviours might have other benefits in their own right, they are unlikely to substantially alter the association between LTL and the risk of major disease outcomes.

## Data sharing

All data used in this study, including telomere length measurements, are available through registration on the UK Biobank.

## Declaration of interests

We declare no competing interests.

## References

[1] Allsopp RC, Vaziri H, Patterson C (1992). Telomere length predicts replicative capacity of human fibroblasts. Proc Natl Acad Sci USA.

[2] Vasa-Nicotera M, Brouilette S, Mangino M (2005). Mapping of a major locus that determines telomere length in humans. Am J Hum Genet.

[3] Broer L, Codd V, Nyholt DR (2013). Meta-analysis of telomere length in 19,713 subjects reveals high heritability, stronger maternal inheritance and a paternal age effect. Eur J Hum Genet.

[4] Factor-Litvak P, Susser E, Kezios K (2016). Leukocyte telomere length in newborns: implications for the role of telomeres in human disease. Pediatrics.

[5] Codd V, Denniff M, Swinfield C (2022). Measurement and initial characterization of leucocyte telomere length in 474,074 participants in UK Biobank. Nat Aging.

[6] Haycock PC, Burgess S, Nounu A (2017). Association between telomere length and risk of cancer and non-neoplastic diseases: a Mendelian randomization study. JAMA Oncol.

[7] Li C, Stoma S, Lotta LA (2020). Genome-wide association analysis in humans links nucleotide metabolism to leukocyte telomere length. Am J Hum Genet.

[8] Codd V, Wang Q, Allara E (2021). Polygenic basis and biomedical consequences of telomere length variation. Nat Genet.

[9] Richter T, von Zglinicki T (2007). A continuous correlation between oxidative stress and telomere shortening in fibroblasts. Exp Gerontol.

[10] Jurk D, Wilson C, Passos JF (2014). Chronic inflammation induces telomere dysfunction and accelerates ageing in mice. Nat Commun.

[11] Valdes AM, Andrew T, Gardner JP (2005). Obesity, cigarette smoking, and telomere length in women. Lancet.

[12] Bateson M, Aviv A, Bendix L (2019). Smoking does not accelerate leucocyte telomere attrition: a meta-analysis of 18 longitudinal cohorts. R Soc Open Sci.

[13] Gielen M, Hageman GJ, Antoniou EE (2018). Body mass index is negatively associated with telomere length: a collaborative cross-sectional meta-analysis of 87 observational studies. Am J Clin Nutr.

[14] Crous-Bou M, Fung TT, Prescott J (2014). Mediterranean diet and telomere length in Nurses' Health Study: population based cohort study. BMJ.

[15] Canudas S, Becerra-Tomás N, Hernández-Alonso P (2020). Mediterranean diet and telomere length: a systematic review and meta-analysis. Adv Nutr.

[16] Mundstock E, Zatti H, Louzada FM (2015). Effects of physical activity in telomere length: systematic review and meta-analysis. Ageing Res Rev.

[17] Valente C, Andrade R, Alvarez L, Rebelo-Marques A, Stamatakis E, Espregueira-Mendes J (2021). Effect of physical activity and exercise on telomere length: systematic review with meta-analysis. J Am Geriatr Soc.

[18] Bycroft C, Freeman C, Petkova D (2018). The UK Biobank resource with deep phenotyping and genomic data. Nature.

[19] Li Y, Pan A, Wang DD (2018). Impact of healthy lifestyle factors on life expectancies in the US population. Circulation.

[20] Khera AV, Emdin CA, Drake I (2016). Genetic risk, adherence to a healthy lifestyle, and coronary disease. N Engl J Med.

[21] Mozaffarian D (2016). Dietary and policy priorities for cardiovascular disease, diabetes, and obesity: a comprehensive review. Circulation.

[22] White IR, Royston P, Wood AM (2011). Multiple imputation using chained equations: issues and guidance for practice. Stat Med.

[23] Lee JJ, Wedow R, Okbay A (2018). Gene discovery and polygenic prediction from a genome-wide association study of educational attainment in 1.1 million individuals. Nat Genet.

[24] Liu M, Jiang Y, Wedow R (2019). Association studies of up to 1.2 million individuals yield new insights into the genetic etiology of tobacco and alcohol use. Nat Genet.

[25] Farzaneh-Far R, Lin J, Epel ES, Harris WS, Blackburn EH, Whooley MA (2010). Association of marine omega-3 fatty acid levels with telomeric aging in patients with coronary heart disease. JAMA.

[26] Dempsey C, Musicha C, Rowlands AV, et al. Causal associations of self-reported walking pace with telomere length on 405,981 middle-aged adults: a UK Biobank study. *Commun Biol* (in press). 10.1038/s42003-022-03323-x PMC9021230 35444173

[27] Nakao T, Bick AG, Taub MA (2022). Mendelian randomization supports bidirectional causality between telomere length and clonal hematopoiesis of indeterminate potential. Sci Adv.

[28] Fry A, Littlejohns TJ, Sudlow C (2017). Comparison of sociodemographic and health-related characteristics of UK Biobank participants with those of the general population. Am J Epidemiol.

